# Needs for care among patients with schizophrenia in six European countries: a one-year follow-up study

**DOI:** 10.1186/1745-0179-2-22

**Published:** 2006-09-11

**Authors:** Viviane Kovess-Masféty, Durk Wiersma, Miguel Xavier, José Miguel Caldas de Almeida, Mauro G Carta, Jacques Dubuis, Elisabeth Lacalmontie, Jacques Pellet, Jean-Luc Roelandt, Francisco Torres-Gonzalez, Berta Moreno Kustner, Dermot Walsh

**Affiliations:** 1 MGEN Foundation for Public Health, EA 4069 University of Paris 5, 3 square Max Hymans, 75748 Paris Cedex 15, France; 2 The Department of Social Psychiatry; University Hospital Groningen, building 32, K5.21, PO Box 30001, 9700 Groningen, The Netherlands; 3 Clinica Universitaria de Psiquiatria e Saude Mental, Faculdade de Ciências Medicas, calçada da Tapada, 155, 1300 Lisboa, Portugal; 4 Istituto di Clinica Psichiatrica, Università degli Studi di Cagliari, viale Merello 22, 09123 Cagliari, Italy; 5 CHS Le Vinatier, 95, boulevard Pinel, 69677 Bron Cedex, France; 6 CHS La Verrière, 78321 Le Mesnil St Denis, France; 7 Service Universitaire de Psychiatrie Adultes, CHU St Etienne, 42055 Saint Etienne Cedex 02, France; 8 Clinique Jérôme Bosch, 104, rue du Général Leclerc, BP 10, 59487 Armentières Cedex, France; 9 Departamento de Psiquiatria, Facultad de Medicina, avenida de Madrid, s/n, 18071 Granada, Spain; 10 Health Research Board, Holbrook House, Holles Street, Dublin 2, Ireland

## Abstract

**Background:**

This article compares needs for care among patients with schizophrenia across six European countries and examines how this relates to the diversity of psychiatric systems in Europe.

**Methods:**

A one-year prospective cohort study was set up. Inclusion criteria for patients were: a clinical lifetime diagnosis of schizophrenia according to ICD-10 (F20) diagnostic criteria for research, age between 18 and 65 years and at least one contact with mental health services in 1993. The patients were assessed for their clinical diagnosis and symptoms using the SCAN interview (*Schedules for Clinical Assessment in Neuropsychiatry*) and the interventions proposed to them were recorded through the systematic use of the NFCAS (*Needs For Care Assessment Schedule*).

**Results:**

438 patients were included and 391 were followed up. The mean age was 38 years, the mean age at onset was 22 years, and 59% were out-patients, 24% in day care and 15% hospitalized. The populations in the different centres were significantly different for almost all the variables: sociodemographic, clinical and social, and the problems identified remained relatively stable over the year. Comparisons highlighted cultural differences concerning the interventions that were proposed. Centres in Italy, Spain and Portugal proposed many interventions even though they were relatively deprived in terms of resources, and the tendency seems to be the reverse for the Northern European countries. On average, one in four patients suffered from needs that were not adequately met by the mental health service in their region. These needs (on average 6 per patient) varied from psychotic symptoms to managing their own affairs. The number of interventions was not correlated to the need status. The availability of community-based treatment, rehabilitation and residential care seems to predict smaller proportions of patients with unmet needs.

**Conclusion:**

There appeared to be a systematic relationship between the availability of community-based mental health care and the need status of schizophrenic patients: the fewer out-patient and rehabilitation services available, the more unmet needs there were.

## Background

This work is part of a collaborative research project. Some results from the project, concerning diversity of team choices, have already been published [1]. The study presented here aims to compare needs for care in patients with schizophrenia across 9 centres in six European countries and to relate this to the diversity of psychiatric systems.

Indeed, there are large differences across these countries mainly because of the diverse historical backgrounds and the various resources available for severely mentally ill patients. Indeed, the deinstitutionalization process has been established at various levels over the last 20 years in most of the European countries. This process has been defined by Bachrach [2] as the replacement of long-stay psychiatric hospitals by smaller, less isolated community-based services, which provide alternatives for the care of the mentally ill. Ideally, community care combined with some kind of rehabilitation and sheltered living accommodation should be developed for chronic psychiatric patients as a response to the gradual closing-down of beds in mental hospitals [3-7]. Two issues seem to be particularly important: achieving continuity of care and the provision of comprehensive care consisting of a wide variety of psychiatric, medical, social, rehabilitative, residential and vocational services.

In the European Union member states, the deinstitutionalization process has been implemented quite diversely. In some countries, the ratio of psychiatric beds per 1,000 inhabitants remains high, whereas in others this ratio is low, either because of an effort to decrease it or because of a lack of availability. It is also important to mention that social benefits provided to those suffering from severe psychiatric disorders are also diverse across countries. Furthermore, the alternatives to long term hospitalisation, like sheltered housing, are also diversely developed, and in some countries the absence of such resources forces patients to live in their family's homes. The relationships between in- and out-patient care systems and the relationships between the psychiatric system and the primary care system are quite diverse, therefore continuity of care is provided in various manners.

A systematic comparison and evaluation of patients' needs and their one-year evolution across EU countries can bring useful information on the process of deinstitutionalization. This idea, reinforced by the results of a workshop and a plan of action [8], led to the setting up of a network of researchers and clinicians ERGOS (*European Research Group On Schizophrenia*) located in 9 centres, in six countries: France (with centres in Lille, Lyon, Saint Etienne and La Verrière), Ireland (Dublin), Italy (Cagliari), the Netherlands (Groningen), Portugal (Lisbon) and Spain (Granada) the main objective being to describe and compare the psychiatric care provided for a group of chronic schizophrenic patients in a circumscribed geographical area in each participating country from South and North Europe. The assessment of the effectiveness of service response required a follow-up of patients to see to what extent changing needs were being met in the course of time. Therefore, the design of the study was prospective and aimed to monitor this process over a period of one year. The results of this study will be reported in this paper which focuses on the evolution over one year of the needs for care in the different centres.

## Methods

This one-year prospective cohort study included patients with a clinical lifetime diagnosis of schizophrenia according to ICD-10 (F20) diagnostic criteria for research [9], aged between 18 and 65 years old and having had at least one contact with the mental health services during the year before inclusion except for the Netherlands where this contact could have occurred long before the study. The selection of patients was conducted on the basis of the clinical diagnosis which had to be confirmed by the use of a standardized interview schedule for present state, the SCAN (*Schedules for Clinical Assessment in Neuropsychiatry*) version 1.0 [10], which also allows for an assessment of lifetime representative episodes of schizophrenia. Clinicians were trained in each country by WHO trainers in their own language and translations were those developed by the WHO.

Patients were randomised for the study independently of whether they were receiving in- or out-patient care. The period from inclusion to first assessment was never greater than six months. The centres did not all start at the same time: e.g. Groningen started in 1992, while Lisbon commenced in late 1994.

### Centres and their settings

A systematic description of the field centres comprised the following: the total population in the catchment area, and the settings and services available to patients with severe mental illness, including the number of in-patient facilities, the number of psychiatric beds, the places in day care and in day hospital, and in community living under professional supervision, and also information on the existence of a structural linkage between in- and out-patient services within the psychiatric system and between the psychiatric services and primary health care. A more extensive description of the centres and their care systems is outside the scope of this paper, but some basic data on mental health care facilities in the catchment area are summarized in Table 1.

**Table 1:** The mental health service system in nine study areas (incl population size) in six countries: hospital beds and places in sheltered accommodation in ratios per 1,000 of total population

	Lille France	Lyon France	St Etienne France	La Verrière France	Dublin Ireland	Groningen Netherlands	Cagliari Italy	Lisbon Portugal	Granada Spain
	84,000	118,000	206,000	NA	254,000	998,000	70,000	280,000	395,000
Beds in general and psychiatric hospitals	.30	.41	.39	NA	.38	2.29	.21	.10	.14
Places in day-care or day-hospitalization	1.00	.21	.47	NA	.59	.74	.00	.04	.06
Places in sheltered accommodation	2.60	.14	.13	NA	.45	.35	.00	.00	.06
Total hospital beds, and places in day-care/hospital and sheltered accommodation	3.90	.76	.98	NA	1.42	3.38	.21	.14	.26

NA Not available

The bed availability ratios varied between 0.10 to 0.41 per 1,000 inhabitants, with the exception of the Dutch centre (2.29), while the ratios of sheltered housing varied from zero in Cagliari and Lisbon to 2.60 in Lille. Little or no day care and day hospitalization were available in three of the nine centres. A ranking of centres on the basis of the total ratios of beds and places yielded three centres as being poorly equipped (Lisbon, Cagliari, Granada), and three as being relatively well equipped (Lille, Groningen and Dublin). The position of La Verrière could not be determined in this respect because of a lack of defined catchment area. Differences between centres existed as to the co-ordination and linkage of in- and out-patient care: teams following the patients in their itinerary through mental health care were present in most centres but lacking in some, particularly in Groningen [11].

### Patients

The selection procedure was similar in all centres except one. Each centre was to prepare a list of all eligible patients in contact with the services during the preceding year and then to randomly select a minimum of twenty patients and preferably fifty from that list. The exception was the Netherlands, which selected patients with significant problems from a 15-year follow-up incidence cohort [12-14].

The first assessment took place at inclusion, as soon as possible after random selection from the list and after written informed consent. In total, approximately 1,100 patients were on the medical "active" files in the nine research centres, of whom 581, i.e. about 50%, were selected. 18 were subsequently excluded since they did not fulfil the diagnostic criteria. Of the 563 remaining patients, 76 (11.5%) could not be contacted or contact was no longer necessary because of a sufficient number of respondents, and 49 (8.7%) refused to cooperate. Thus, about 75% of the patients selected, i.e. 438, were assessed (see Table 2): 433 completed the PHSD (*Past History and Sociodemographic Description schedule*), 430 had a NFCAS (*Needs For Care Assessment Schedule*) assessment at entry and 391 were followed over one year.

**Table 2:** The selection and inclusion of the patients in the 9 centres

	Lille France	Lyon France	La Verrière France	St Etienne France	Dublin Ireland	Cagliari Italy	Groningen Netherlands	Lisbon Portugal	Granada Spain	Total
Randomly selected from the files and fulfilling the diagnostic criteria	73	53	33	98	78	25	48	50	105	563
No contact possible or needed any longer	2	5	3	34	10	2	-	-	20	76
Refusals	21	3	3	14	4	3	-	-	1	49
Included and assessed	50	45	27	50	64	20	48	50	84	438
PHSD	46	44	27	50	64	20	48	50	84	433
NFCAS at baseline	46	44	27	50	64	20	48	50	81	430
NFCAS at time 2	42	40	25	46	52	18	38	50	80	391

The characteristics of the patient population in each centre are set out in Table 3. For the sake of simplicity, not all categories of a characteristic (e.g. living situation, education) are specified and therefore percentages do not add up to 100%. Distributions of gender and marital status differed between the centres (p < .05) while age distribution did not.

**Table 3:** Characteristics of patient populations at entry for nine study areas in six countries in percentages or means

	Lille France	Lyon France	La Verrière France	St Etienne France	Dublin Ireland	Groningen Netherlands	Cagliari Italy	Lisbon Portugal	Granada Spain	Total	p
Number of patients	46	44	27	50	64	48	20	50	84	433	
Male (%)	65	73	63	78	61	54	80	82	75	70	S
Mean Age (sd)	37 (9.2)	38 (9.8)	38 (10.8)	37 (9.5)	43 (10.5)	40 (7.4)	33 (10)	34 (8.4)	35 (8.7)	38 (9.7)	NS
Never Married (%)	69	83	89	82	67	58	80	86	82	77	S
Sheltered accommodation* (%)	13	27	11	10	3	30	10	0	1.2	10	S
Private accommodation (%)	78	73	89	86	81	71	90	100	99	86	
No professional training (%)	41	50	37	24	67	50	50	28	74	50	NS
University education (%)	0	14	30	4	0	8	10	6	4	6	
Regular wages (%)	9	10.5	11	4	12.5	15	0	16	11	11	S
On social benefit or pension (%)	84	82	78	94	77	67	10	28	64	67	S

* Homeless: 15.6% Dublin, 4% Saint Etienne, 0% elsewhere

There is marked variation in these characteristics across centres. Very few patients in Cagliari, Lisbon and Granada were living alone, while the reverse was true in Lille, La Verrière and Groningen. Fewer patients were in receipt of social benefits in Cagliari and Lisbon than elsewhere because of the lack of a social security system. Patients in Dublin were less educated than elsewhere, while in all centres, except for Dublin, about 9% had a university degree. La Verrière had a high proportion of patients with a university degree due to its specific care system and its recruitment.

The time of onset of psychosis varied between the ages of 18 and 26 years (22 years on average), while the first contact with mental health care occurred up to two years later. Nearly all patients were on neuroleptic medication, except for those in the Groningen centre because a relatively large number were out of care at time of assessment (most had used medication in the preceding two years). Most centres had some proportion of patients assessed during their stay in hospital (15% on average, but none in Lisbon and only a few in Dublin and Granada).

### Needs for care

The key instrument was the NFCAS second version, developed by Brewin and Wing [15], and described in great detail by Brewin et al. [16] (see also [5]). Inter-rater reliability was tested [13,16-20] and was generally found to be good to excellent. The NFCAS assesses 20 problems in clinical and social functioning For the purpose of this study, two items on the use of alcohol and drugs were added. The investigator used a list of items of care relevant to each identified problem after which he had to determine the need status with regard to each problem. A need existed if there was a problem in an area, i.e. the level of functioning was below or might fall below some minimum specified level, and there was a potential remedial intervention or form of care.

The NFCAS covers eleven areas of clinical state and eleven areas of social functioning. Examples of clinical problems are psychotic symptoms, negative symptoms, and physical disease. Social problems relate to skills and abilities for self-care and household management. The rating of a specific intervention is determined by its appropriateness, effectiveness and acceptability to the patient. The ratings of both the level of functioning and the interventions provide the following results: no need (no problem), met need (problem is adequately taken care of), unmet need for assessment (an assessment is needed), unmet need for treatment (an effective and acceptable intervention is not delivered), and non-meetable need (a potentially effective intervention is not available or is being refused by the patient). This is the primary need status which is expected to generate the greatest professional consensus. Three additional ratings are made: (a) overprovision referring to continuation of an item of care (e.g. medication) for which there is no longer any reason, (b) future needs referring to delivery of an item of care for which intervention will become appropriate, and (c) possible needs referring to the lack of performance of a social skill for which some kind of intervention may become appropriate. The need status of patients was analyzed according to the following categories: (a) patients with no needs or met needs only, (b) patients with no needs, met needs and at least one non-meetable need, but without unmet needs, and (c) patients with at least one unmet need.

### Training

The raters from the nine centres were trained in the use of the SCAN and the NFCAS by different trainers (see acknowledgments). The inter-rater reliability of the NFCAS was based on two series of written case vignettes and yielded an overall kappa of .56 and 88% agreement for clinical problems and 84% agreement for social problems [13]. In all centres, ratings were based on discussions on written or verbal case descriptions between two or more interviewers or in team-meetings.

### Analysis

The results were analyzed by means of descriptive and correlation statistics (Spearman rank correlation). Differences between centres were tested by chi square and difference of means tests.

## Results

### Prevalence of needs for care

The main problems at the time of first assessment exploring needs among at least circa one third of the patient population were (Table 4 and Table 5): psychotic symptoms (87%), slowness and under-activity (58%), dyskinesia or other side effects of medication (33%) as regards clinical needs, and occupational skills (42%), managing own affairs (41%), managing money (35%) and problems in carrying out household chores (33%) as regards social needs. Dementia or organic psychosis, use of drugs or alcohol, lack of basic academic skills, and use of public transport were areas of much lesser importance (<10%).

**Table 4:** Frequency of individual clinical (A) needs at inclusion (score 1 and 2) (%)

	Lille	Lyon	St-Etienne	La Verrière	Dublin	Groningen	Cagliari	Lisbon	Granada	Total	p
	N = 46	N = 44	N = 50	N = 27	N = 64	N = 48	N = 20	N = 50	N = 81	N = 430	
Psychotic symptoms	80.4	93.2	100.0	92.6	68.8	68.8	75.0	100.0	100.0	87.4	.000
Slowness, under-activity	37.0	65.9	68.0	81.5	45.3	27.1	75.0	62.0	71.6	57.7	.000
Side effects, dyskinesia	28.3	43.2	40	51.9	26.6	12.5	30	48.0	27.2	32.8	.002
Neurotic symptoms	19.6	43.2	6.0	22.2	9.4	33.3	30.0	16.0	23.5	21.4	.000
Dementia	0.0	0.0	0.0	0.0	1.6	0.0	0.0	0.0	0.0	0.2	NS
Physical symptoms	13.0	31.8	42.0	33.3	6.3	4.2	15.0	14.0	7.4	16.7	.000
Dangerous behaviour	15.2	29.5	32.0	22.2	15.6	14.6	40.0	24.0	19.8	22.1	NS
Embarrassing behaviour	15.2	27.3	12	11.1	9.4	12.5	55.0	40.0	24.7	21.2	.000
Distress	21.7	38.6	34.0	7.4	12.5	22.9	20.0	28.0	38.5	26.5	.003
Alcohol	13.0	4.5	10.0	11.1	10.9	10.4	0.0	16.0	21.0	12.3	NS
Drugs	0.0	4.5	4.0	3.7	4.7	4.2	10.0	6.0	7.4	4.9	NS

**Table 5:** Frequency of individual social (B) needs at inclusion (%) (score 1, 2, 3)

	Lille	Lyon	St-Etienne	La Verrière	Dublin	Groningen	Cagliari	Lisbon	Grenada	Total	p
	N = 46	N = 44	N = 50	N = 27	N = 64	N = 48	N = 20	N = 50	N = 81	N = 430	
Personal hygiene	17.4	36.4	26.0	18.5	40.6	33.3	35.0	10.0	34.6	28.8	.008
Shopping	4.3	18.2	14.0	7.4	17.2	8.3	50.0	38.0	42.0	22.6	.000
Getting meals	26.1	36.4	16.0	29.6	10.9	8.3	45.0	32.0	42.0	26.5	.000
Household chores	30.4	43.2	26.0	25.9	34.4	18.8	35.0	32.0	43.2	33.0	-
Public transports	6.5	11.4	10.0	18.5	9.4	4.2	30.0	6.0	17.3	11.4	.038
Public amenities	21.7	22.7	30.0	44.4	20.3	6.3	35.0	10.0	24.7	22.1	.003
Education	2.2	9.1	6.0	3.7	6.3	2.1	15.0	0.0	6.2	5.1	-
Occupation	63.0	31.8	16.0	44.4	39.1	27.1	65.0	58.0	44.4	41.6	.000
Communication skills	6.5	4.5	8.0	7.4	39.1	18.8	45.0	20.0	29.6	20.5	.000
Managing money	21.7	34.1	32.0	18.5	26.6	27.1	50.0	76.0	34.6	35.3	.000
Managing own affairs	67.4	61.4	40.0	33.3	18.8	27.1	65.0	36.0	39.5	40.7	.000

Centres differed one from the other for the prevalence of problems in specific areas. For example, neurotic symptoms of anxiety and depression were relatively common in Lyon, Cagliari and Groningen, while physical health problems were common in three of the four French centres. The occurrence of aggressive behaviour was perceived as a problem in Cagliari and socially unacceptable behaviour was problematic in Cagliari and Lisbon. Needs regarding occupational skills and activities were frequently noted in Lille and Lisbon; this applied particularly to difficulties in managing money in Lisbon, and also concerned the inability of patients to manage their own affairs in Lille, Lyon and Cagliari.

Generally, patients had on average 6 problems, clinical needs being slightly more frequent (mean 3.1) than social needs (mean 2.9.). Patients in Groningen and Dublin had fewer problems with total scores of 3.9 and 4.7 respectively and patients in Cagliari and Granada scored highest with 8.2 and 6.9 problems respectively.

Most problems (60–80%) appeared to be adequately met by mental health care (Table 6, Table 7). When this was not the case, social needs were twice as often unmet as clinical needs, particularly in centres like Granada, Cagliari, Lisbon, Dublin and Lyon where the ratio of met to unmet needs was less than 3 to 1. On average 9% of clinical needs and 22% of social needs were not meetable either because of refusal by the patient or on account of absence of effective interventions. Centres which ranked high on the met: unmet ratio (e.g. > 5:1) were the Dutch centre and the four French centres. Lille and La Verrière were very effective in fulfilling the needs of their patients, while Cagliari, Granada and Lisbon performed much less well.

**Table 6:** Clinical and social needs – status evolution (1)

		Lille France		Lyon France		La Verrière France		St Etienne France		Dublin Irland		Cagliari Italy		Groningen Netherlands		Lisbon Portugal		Granada Spain		Total		p	
		Inc	end	Inc	end	Inc	end	Inc	end	Inc	end	Inc	end	Inc	end	Inc	end	Inc	end	Inc	end	Inc	end
Clinical	Needs	**2.43**	2.13	**3.81**	3.30	**3.37**	2.93	**3.78**	3.08	**2.11**	1.83	**3.55**	2.25	**2.17**	1.52	**3.54**	3.10	**3.48**	3.14	**3.09**	2.63	**0.00**	0.00
	Met needs	**1.76**	1.65	**2.95**	2.68	**3.11**	2.67	**2.74**	2.18	**1.63**	1.63	**2.40**	1.80	**1.23**	0.79	**2.72**	2.44	**2.67**	2.37	**2.31**	2.03	**0.00**	0.00
	Unmet needs	**0.15**	0.04	**0.18**	0.00	**0.00**	0.00	**0.44**	0.36	**0.09**	0.08	**0.55**	0.20	**0.29**	0.38	**0.52**	0.30	**0.22**	0.33	**0.26**	0.21	**0.00**	0.16
	Non-meetable needs	**0.39**	0.41	**0.59**	0.48	**0.11**	0.19	**0.52**	0.50	**0.23**	0.08	**0.40**	0.10	**0.44**	0.25	**0.10**	0.26	**0.28**	0.22	**0.34**	0.28	**0.20**	0.12
Social	Needs	**2.67**	2.67	**3.09**	2.18	**2.52**	2.52	**2.24**	2.18	**2.63**	2.56	**4.70**	3.30	**1.81**	1.69	**3.18**	2.50	**3.58**	3.80	**2.88**	2.65	**0.00**	0.01
	Met needs	**1.70**	2.00	**2.09**	1.52	**1.67**	1.74	**1.48**	1.60	**1.56**	1.94	**1.90**	1.35	**1.17**	0.98	**2.04**	1.76	**1.62**	1.70	**1.67**	1.64	**NS**	NS
	Unmet needs	**0.39**	0.09	**0.59**	0.16	**0.30**	0.11	**0.56**	0.32	**0.36**	0.15	**1.75**	1.20	**0.15**	0.21	**1.00**	0.58	**1.51**	1.11	**0.74**	0.46	**0.00**	0.00
	Non-meetable needs	**0.85**	0.57	**0.61**	0.55	**0.63**	0.70	**0.72**	0.42	**0.52**	0.29	**1.10**	0.70	**0.48**	0.50	**0.14**	0.18	**0.74**	1.05	**0.61**	0.57	**NS**	0.03
Total	Needs	**5.11**	4.80	**6.91**	5.48	**5.89**	5.44	**6.02**	5.26	**4.73**	4.38	**8.25**	5.55	**3.98**	3.21	**6.72**	5.60	**7.06**	6.94	**5.97**	5.28	**0.00**	0.00
	Met needs	**3.46**	3.65	**5.05**	4.20	**4.78**	4.41	**4.22**	3.78	**3.19**	3.58	**4.30**	3.15	**2.40**	1.77	**4.76**	4.20	**4.28**	4.07	**3.97**	3.67	**0.00**	0.00
	Unmet needs	**0.54**	0.13	**0.77**	0.16	**0.30**	0.11	**1.00**	0.68	**0.45**	0.23	**2.30**	1.40	**0.44**	0.58	**1.52**	0.88	**1.73**	1.44	**1.00**	0.67	**0.00**	0.00
	Non-meetable needs	**1.24**	0.98	**1.20**	1.02	**0.74**	0.89	**1.24**	0.92	**0.75**	0.37	**1.50**	0.80	**0.92**	0.75	**0.24**	0.44	**1.02**	1.27	**0.95**	0.85	**0.20**	0.37

Met needs + unmet needs + non-meetable needs < total needs because «9» (non applicable) are excluded.

**Table 7:** Clinical and social problems, and met and unmet needs for 9 study areas in 6 countries, percentages and ratios

	Lille N = 46	Lyon N = 44	St Etienne N = 50	La Verrière N = 27	France	Dublin N = 64	Groningen N = 48	Northern Europe	Cagliari N = 20	Lisbon N = 50	Granada N = 81	Southern Europe	Total N = 430
Perc. clinical met needs	75.80	80.52	79.80	93.67		75	56.88		69.47	79.73	79.01		76.96
Perc. clinical unmet needs	70.10	4.50	9.30	0,00		5.60	15.60		15.00	14.3	7.1		8.60
Perc. clinical non-meetable needs	13.08	13.14	9.43	2.20		9.69	18.06		9.82	2.37	6.70		9.42
Perc. social met needs	67.47	68.94	75.14	66.20		51.92	60.73		35.97	67.03	41.34		59.67
Perc. social unmet needs	25.76	17.77	34.68	0.90		16.26	12.50		41.57	29.08	48.59		27.12
Perc. social non-meetable needs	27.49	20.53	29.26	27.33		26.54	24.81		23.38	0.00	17.89		21.61
Ratio met/unmet clinical needs	11.57	16.25	6.22		**11.67**	17.33	4.21	**8.15**	4.3	5.2	12	**7.27**	8.88
Ratio met/unmet social needs	4.33	3.53	2.64	5.62	**3.61**	4.34	8	**5.2**	1.08	2.1	1.07	**1.30**	2.25
Ratio met/unmet all needs	6.36	6.52	4.22	16.12	**6.16**	7.03	5.47	**6.38**	1.86	3.13	2.47	**2.56**	3.98
**Percentage of patients with clinical needs**	63.0	56.8	60.0	88.9		78.1	52.1		50.0	58.0	70.4		64.9
no/met needs only	21.7	27.3	10.0	11.1		14.1	20.8		25.0	8.0	9.9		15.3
no/met/non-meetable needs only at least one unmet need	15.2	15.9	30.0	0.0		7.8	27.1		25.0	34.0	19.8		19.8
**Percentage of patients with social needs**	32.6	47.7	48.0	44.4		51.6	66.7		20.0	52.0	38.3		46.0
no/met needs only	43.5	22.7	20.0	33.3		25.0	20.8		30.0	2.0	8.6		20.7
no/met/non-meetable needs only at least one unmet need	23.9	29.5	32.0	22.2		23.4	12.5		50.0	46.0	53.1		33.3
**Percentage of patients with needs (total)**	26.1	36.4	34.0	40.7		45.3	43.8		15.0	42.0	29.6		35.8
no/met needs only	39.1	27.3	16.0	37.0		25.0	25.0		35.0	6.0	8.6		21.6
no/met/non-meetable needs only at least one unmet need	34.8	36.4	50.0	22.2		29.7	31.3		50.0	52.0	61.7		42.6

The need status of patients enabled estimation of the proportion of the population of patients for whom the mental health care system needs to ensure wider provision. This illustrates the size of the problem in a different way. About one third of the patients had only met needs. One in five also had non-meetable needs (without unmet needs); in Lille, however, this proportion was nearly one in two and may reflect an unwilling attitude on the part of patients who refuse to utilize a variety of rehabilitative services. It appeared further that a substantial proportion of patients in all study areas were suffering from unmet needs: on average four out of every ten patients. This proportion varied from 19% in La Verrière to 100% in Cagliari.

### Evolution over one year

Over one year, there was little variation in the patients' symptoms. Dangerous and embarrassing behaviour decreased and problems concerning hygiene, shopping and occupation seemed to be less frequent.

The number of needs decreased slightly during this period. The average number of clinical needs decreased more than the average number of social needs.

### The availability of services and the fulfilment of needs

The relationship between the proportion of patients with unmet needs and the availability of mental health services was investigated. The ranking of the eight centres (except for La Verrière) from high to low in the ratio of hospital beds, of places in day care and day hospitals, the sheltered housing ratio and the proportion of patients with an unmet need provides a rough indication of an association between these two variables. There appeared to be a statistically significant correlation between the proportion of patients with an unmet need and the ratio of day care and day hospital places (Spearman correlation coefficient r = -.77; p = .015) and community residential places (r = -.82; p = .009), but not with the bed ratio (r = -.50; p = 0.17). This means that centres with relatively more day-care and day-hospital beds and sheltered places "produced" less unmet needs among their population of schizophrenic patients. Unmet clinical needs were not related to the number of either beds or community places in contrast to unmet social needs: the correlation between the total number of beds and day or sheltered places in mental health care and the number of unmet social needs was highly statistically significant (r = -.90; p = .001), but this was not the case with clinical unmet needs (r = -.40; p = .29).

## Discussion

This report outlines a study conducted in nine centres in six European countries, among which several centres had no prior experience in research and it was the professionals involved in patient care who also had to act as researchers. The experience of this study shows that the NFCAS proves to be a useful tool for standardized assessment of needs among schizophrenic patients cared for in community-based mental health care settings. The validity and intra-centre reliability of the NFCAS were subject to regular checks. Inter-centre agreement, however, did not reach a high level of reliability, which limits the generalisation of the study results. This relates partly to unclear instructions in the NFCAS manual and descriptive response categories that even the original trainers who were consulted could not resolve, or disagreed on among themselves. Disagreement partly stemmed from the use of different criteria based on differential standards and cultural values. For example, a recurring theme concerned the cultural differences between Southern and Northern European attitudes towards men carrying out household chores.

The selection of the samples aimed at representing the patient populations treated in the different catchment areas. However, long stay patients in some centres (but not the majority) were excluded, as were those who had contact with the general practitioner only, or cases not in care at all (e.g. homeless). It is not unlikely that, to some extent, the more seriously ill patients were not adequately represented in this study, as well as possibly less severe cases in contact with the general practitioner. The samples were not homogeneously and uniformly selected across all centres. Selection procedures differed between centres, which resulted in somewhat different study populations. Also, the small numbers of patients selected in the centres at Cagliari (20) and La Verrière (27) restrict the possibility of generalising the findings. Drop-out due to refusal (most of them in one centre) or for other reasons, was limited to 25%. However, this 400-subject sample provides a fairly accurate image of the "average" or "modal" schizophrenic patients in regular contact with mental health services in nine centres across six European countries. Typical subjects were males, between 35 and 43 years old who had been ill for about 15 years, unmarried, living alone or with parents, unemployed and dependent on social benefits. The last point was not the case in the Southern European countries where patients relied on their families for socio-economic support.

The preponderance of males in most centres may partly reflect better long term outcome for female patients as reported from the International Pilot Study of Schizophrenia [21].

Patients suffered from a variety of problems of clinical and social nature. There was a general absence of needs in the areas of organic psychosis, education and abuse of alcohol and drugs. The overall differences in need profiles between the nine centres are relatively small: on average six problems, the fewest being 3.9 in Groningen and the most 8.4 in Cagliari and 7.3 in Granada. The mean number of needs in other studies in England, Canada, Italy and Finland using the same instrument (NFCAS) varied between 3.8 among schizophrenic out-patients in Verona to 9.1 among residents in hostels in Oxford and 10.6 among long-stay patients in South Glamorgan (14). These high scores probably reflect a selected group of more seriously ill patients with many physical problems too.

Unmet clinical and social needs were rather ubiquitous. Unmet clinical needs were found in equal proportions in all centres and the ratios of met to unmet needs were rather favourable (mostly > 5:1). However, the Southern European centres (Cagliari, Lisbon and Granada) had higher numbers of unmet social needs, unfavourable ratios of met to unmet needs (mostly < 2:1) and higher percentages of patients with unmet needs than in the other centres. Although most French centres had high ratios of met to unmet needs (especially clinical needs), the number of patients with unmet needs was still substantial between 19% and 50%. Even in a model, strongly community-oriented area, like Lille, patients with unmet needs were not exceptional. It was remarkable that a large proportion of non-meetable needs was found in this centre, due to refusal on the part of patients. This kind of research, therefore, has the potential to raise awareness concerning the comprehensiveness and flexibility of services, particularly if carried out in close collaboration with the clinical staff.

A feature of the study was the statistically significant relationship between the availability of out-patient care, rehabilitative facilities and sheltered housing and relatively lower proportions of patients with unmet needs. This relationship holds particularly for the number of social needs that were not adequately fulfilled. Clinical needs were met independently of the number of beds or the availability of other mental health services. Centres in the Southern European countries performed worse in this respect due to a lack of alternative structures for day care and day treatment, and especially for rehabilitation and housing, which therefore created a situation of need. Consequently, families had to cope with the emotional and financial burden of patient with schizophrenia. Sheltered housing and financial support had to come from relatives instead of from public health or social services. Although integration and linkage within the psychiatric system and with primary care in these centres was generally stronger than in some Northern European centres, the shortage of adequate resources apparently prevented the meeting of social needs.

## Conclusion

Nine research centres, mostly based on local mental health services in France, the Netherlands, Ireland, Italy, Portugal and Spain successfully assembled a cohort of schizophrenic patients and carried out a standardized assessment of their clinical and social needs. Out of 400 randomly selected patients in all centres, on average one in four suffered from needs that were not adequately met by the mental health service in their region. These needs varied from psychotic symptoms to managing their own affairs. Unmet needs occurred in all centres and in all kinds of care systems, even in strongly community-oriented systems with structural linkages to primary care. It is uncertain if this is due to inadequacies in community care, insufficient funding of resources, lack of co-ordination between medical and social services, or other reasons. Further research in this area is needed. There appeared to be a systematic relationship between the availability of community-based mental health care and the need status of patients: the fewer the out-patient and rehabilitation services, the more unmet needs there were. The number of beds was not predictive of the need status, and the number of interventions was not correlated to it. Further research in this area is needed in greater detail, such as the methods implemented to cater for needs, the role of the family and the role of specific interventions by the health care services.

## Abbreviations

ICD International Classification of Diseases

SCAN Schedules for Clinical Assessment in Neuropsychiatry

PHSD Past History and Sociodemographic Description schedule

NFCAS Needs For Care Assessment Schedule

## Competing interests

The author(s) declare that they have no competing interests.

## Authors' contributions

VKM was involved in the design of the study, statistical analysis and drafting of the manuscript. DWi, MX, JMCA, MGC, JD, EL, JP, JLR, FTG, BMK and DWa participated in the design of the study and data collection, and provided comment on the content of the manuscript.

**Table 8:** Number of interventions per patient in out-patient care over one year (means)

	Sheltered accommodation	Activities of daily living	Social intervention	Biological-psychiatric intervention	Crisis management	Somatic-medical care	Sheltered work	Assessment	Psychotherapeutic intervention	Occupational therapies	Total number of interventions
La Verrière	0.00	3.46	3.16	6.89	3.10	1.86	1.15	14.06	39.70	40.86	114.25
Granada	0.16	6.04	0.45	8.41	0.00	2.02	6.21	2.71	1.19	3.93	31.11
Lyon	0.00	2.57	2.49	7.11	0.03	0.71	1.27	1.06	13.74	14.53	43.50
Lisbon	0.00	0.20	0.00	17.92	0.06	0.02	9.14	1.14	6.22	3.34	38.04
Cagliari	0.05	4.77	0.17	40.14	0.16	0.50	0.00	9.56	2.89	0.10	58.33
Dublin	0.04	6.90	1.14	25.64	0.87	0.19	8.24	6.49	0.23	31.73	81.47
Average	0.52	5.92	2.53	18.21	1.30	1.00	4.62	6.66	13.01	15.47	69.24
